# P05.57. Developing good practice guidelines in the treatment of polycystic ovary syndrome with Chinese herbal medicine: a Delphi study

**DOI:** 10.1186/1472-6882-12-S1-P417

**Published:** 2012-06-12

**Authors:** L Lai, A Flower, G Lewith, M Moore

**Affiliations:** 1 University of Southampton, Southampton, United Kingdom

## Purpose

Polycystic ovary syndrome (PCOS) is the most common female endocrine disorder, affecting 6-18% of women of reproductive age. Irregular periods are a hallmark symptom of PCOS and whilst Chinese herbal medicine (CHM) has historically been used as an effective treatment for irregular periods, this requires further investigation in randomised controlled trials (RCTs). To ensure that the methods used within an RCT reflects Chinese herbal methods used in clinical practice, a study was proposed to establish good practice guidelines in the treatment of PCOS through achieving consensus amongst a group of Chinese medicine herbalists.

## Methods

The Delphi method was used involving in-depth interviews with a purposive sample of 11 expert Chinese medicine herbalists. Interview data was analysed using thematic and framework analysis to formulate Delphi questionnaire items. Experts were then distributed the questionnaire online and asked to rate their agreement with each questionnaire item on a 7-point Likert scale. Consensus was defined *a priori* as a mean Likert scale score of 5 or more. Questionnaire items not reaching consensus were re-distributed to experts for re-consideration via a second and a third and final round of questionnaires where necessary.

## Results

Preliminary results suggest that consensus exists amongst experts regarding common diagnostic categories, treatment strategies used, appropriate dosage and appropriate duration of treatment to observe a clinical effect.

## Conclusion

The preliminary results suggest that consensus can be achieved at least within the core aspects of Chinese herbal medicine treatment for PCOS. This methodology is vital in the development of rigorous RCTs for CHM and the outcome and principles of good practice guidelines for PCOS will be presented at the conference.

